# Interleukin-6 mediates resistance to PI3K-pathway–targeted therapy in lymphoma

**DOI:** 10.1186/s12885-019-6057-7

**Published:** 2019-10-10

**Authors:** Joo Hyun Kim, Won Seog Kim, Chaehwa Park

**Affiliations:** 1 Department of Health Sciences and Technology, Samsung Advanced Institute for Health Sciences and Technology, Sungkyunkwan University, Seoul, 06351 Korea; 2 Division of Hematology and Oncology, Department of Medicine, Samsung Medical Center, Sungkyunkwan University School of Medicine, Seoul, 06351 Korea; 3 Research Institute for Future Medicine, Samsung Medical Center, Sungkyunkwan University School of Medicine, Seoul, 06351 Korea

**Keywords:** Lymphoma, PI3K, Copanlisib, Duvelisib, Drug resistance, IL-6

## Abstract

**Background:**

The phosphoinositol 3-kinase (PI3K) pathway is associated with poor prognosis of hematologic malignancies, providing a strong rationale for the use of PI3K inhibitors in the treatment of malignant lymphoma. However, development of resistance limits the use of PI3K inhibitors in lymphoma patients.

**Methods:**

We established copanlisib (pan-PI3K inhibitor)-resistant B-cell lymphoma and duvelisib (PI3Kδ and -γ inhibitor)-resistant T-cell lymphoma cell lines. The cytokine array and the phospho-kinase array were used to identify up-regulated proteins in the resistant cells. Cytokine expression and phospho-kinase levels were examined by ELISA and Western blot analysis, respectively. Cell proliferation capabilities were measured by using CCK-8 kit and colony formation assay. The effects of inhibitors on apoptosis were detected using an Annexin V-FITC Apoptosis Detection Kit and a flow cytometry system. The underlying mechanisms were studied by transfecting recombinant plasmids or siRNA into lymphoma cell lines. Cells were transiently transfected using the Amaxa electroporation system. We evaluated the effects of PI3K inhibitor alone and in combination with JAK inhibitor (BSK805) on lymphoma proliferation and signaling pathway activation.

**Results:**

Cytokine arrays revealed upregulation of interleukin (IL)-6 in both copanlisib- and duvelisib-resistant cell lines. Phosphorylated STAT5, AKT, p70S6K and MAPK were increased in copanlisib-resistant B-cell lymphoma cells, whereas phosphorylated STAT3 and NF-κB were increased in duvelisib-resistant T cell lymphoma cells. Conversely, depletion of IL-6 sensitized both resistant cell lines, and led to downregulation of phosphorylated STAT3 and STAT5 in copanlisib- and duvelisib-resistant cells, respectively. Moreover, combined treatment with a JAK inhibitor (BSK805) and a PI3K inhibitor circumvented the acquired resistance to PI3K inhibitors in lymphoma, and concurrent inhibition of the activated pathways produced combined effects.

**Conclusions:**

IL-6–induced STAT3 or STAT5 activation is a critical mechanism underlying PI3K inhibitor resistance in lymphoma, supporting the utility of IL-6 as an effective biomarker to predict therapeutic response to PI3K inhibitors.

**Electronic supplementary material:**

The online version of this article (10.1186/s12885-019-6057-7) contains supplementary material, which is available to authorized users.

## Background

Non-Hodgkin lymphomas are a heterogeneous group of cancers—many of which are aggressive—comprising B lymphocytes, T lymphocytes and natural killer (NK) lymphocytes [1]. The phosphoinositide 3-kinase (PI3K) signaling pathway is frequently activated in many cancers and has been shown to regulate numerous biological activities, including cellular growth, survival, and proliferation [2, 3]. It has also been shown that overexpression of PI3K isoforms is a predictor of poor prognosis and is also a cause for relapse and therapy resistance [4]. PI3Ks are divided into three classes—I, II, and III— the first of which includes PI3Kα, β, γ, and δ [5]. Of the available PI3K inhibitors, copanlisib is a potent, reversible pan-class I PI3K inhibitor with predominant activity against PI3K-δ and PI3K-α isoforms [6]. In preclinical studies, copanlisib monotherapy demonstrated clinically meaningful responses in patients with relapsed or refractory malignant lymphoma [7–9]. Duvelisib is a small-molecule dual inhibitor of PI3K-δ and PI3K-γ [10] that was previously found to inhibit both PI3K/AKT and BCR (B-cell receptor) signaling pathways [11, 12]. Clinical studies of duvelisib in indolent non-Hodgkin lymphoma and chronic lymphocytic leukemia (CLL) have shown effective clinical activity [13, 14]. Nevertheless, PI3K inhibitor monotherapy results in a low frequency of complete responses, and patients treated with the PI3K inhibitor idelalisib eventually develop resistance owing to activation of NF-κB (nuclear factor kappa-light-chain-enhancer of activated B cells) and mTOR (mammalian/mechanistic target of rapamycin) pathways in activated B cell-like diffuse large B-cell lymphoma (ABC DLBCL) [14–16]. It was recently shown that the PI3K inhibitors, copanlisib and duvelisib, are effective against DLBCL and relapsed/refractory T-cell lymphoma, respectively [17, 18]. IL-6 is a cytokine that is important in controlling the survival, proliferation, population expansion, and maturation of B and T cells. In addition, IL-6 modulates effector cytokine production by B and T cells [19], and also plays an important role in activating several pro-oncogenic signaling pathways in cancer [20, 21].

In the current study, we established copanlisib-resistant B-cell lines and a duvelisib-resistant T-cell line, and investigated PI3K inhibitor resistance mechanisms in these cells. Our results demonstrate that IL-6 overexpression induces PI3K resistance through activation of STAT (signal transducer and activator of transcription) pathways.

## Methods

### Cell lines, culture conditions, transfection, and inhibitors

The T-cell lines, H9, H9/HTLV, HH, HUT78, MJ, Jurkat and SR786, and B cell lines, BJAB (BL), OCI-Ly1 (GCB-DLBCL), Riva (ABC-DLBCL), SU-DHL2 (ABC-DLBCL) and U2932 (ABC-DLBCL), were used in this study. H9, H9/HTLV, HH, HUT78, MJ and SU-DHL2 cells were purchased from American Type Culture Collection (Rockville, MD, USA), and Riva and U2932 cells were purchased from Leibniz-Institut DSMZ-Deutsche Sammlung von Mikroorganismen und Zellkulturen GmbH (Braunschweig, Germany). Jurkat and BJAB cells were kindly provided by Dr. H. Y. Yoo (Sungkyunkwan University, Seoul, Korea), and SR786 and OCI-Ly1 were kindly provided by Dr. Y. K. Jeon (Seoul National University Hospital, Seoul, Korea). Cell lines were cultured in RPMI-1640 medium (H9, H9/HTLV, HH, Jurkat, SR786, BJAB, Riva, SU-DHL2 and U2932) or Iscove’s Modified Dulbecco’s Medium (HUT78, MJ and OCI-Ly1) supplemented with 10% or 20% (HUT78, MJ and OCI-Ly1 cells only) heat-inactivated fetal bovine serum (FBS), penicillin, and streptomycin (Gibco-BRL, Grand Island, NY, USA) in a humidified 5% CO_2_ atmosphere. All cell lines were tested for Mycoplasma and characterized by STR profiling as indicated in the DSMZ online. Cell lines with acquired duvelisib or copanlisib resistance, termed HH-duvel-R and Ly1-copan-R, respectively, were generated by exposing the respective parental cells to progressively increasing concentrations of the corresponding inhibitor for 4 weeks. Small interfering RNAs (siRNAs) were purchased from Santa Cruz Biotechnology (Santa Cruz, CA, USA), and an expression plasmid for constitutively active STAT3 was kindly provided by Dr. C. Park (Samsung Medical Center, Seoul, Korea). Cells were transiently transfected using the Amaxa electroporation system (Amaxa, Gaithersburg, MD). Copanlisib, duvelisib, BSK805, U0126 and velcade were purchased from Selleck Chemicals (Houston, TX, USA), and IL-6 was purchased from Peprotech (Rocky Hill, NJ, USA).

### Assessment of cell viability

Drug effects on cell viability were monitored using trypan blue staining or the Cell Counting Kit-8 reagent (CCK-8) viability assay. For CCK-8 assays, cells were incubated for 48 h at 37 °C in triplicate in a 96-well plate (final volume, 0.1 mL) in the presence or absence of the indicated test samples, followed by addition of 20 μL of CCK-8 reagent (Dojindo Laboratories, Kumamoto, Japan) to each well. After 2-h incubation at 37 °C, optical density (OD) at 450 nm was measured using a 96-well multiscanner autoreader. Cell viability was expressed as a percentage (OD of the experimental sample/OD of control). Viable cells were also determined using trypan blue exclusion assays. For these assays, cells were suspended in a 0.4% trypan blue solution (1:1), loaded onto a hemocytometer, and counted. The calculated percentage of unstained cells represented the percentage of viable cells.

### Cytokine and phospho-kinase arrays

This experiment was performed using proteins collected from conditioned media. The detected IL-6 represented the level of extracellular secretion. Parental cells and cells resistant to the PI3K inhibitors, copanlisib or duvelisib, were collected and applied to a human XL cytokine array (R&D Systems, Inc., Minneapolis, MN, USA) and a human XL phospho-kinase array (R&D Systems) according to the manufacturer’s instructions.

### Elisa

IL-6 protein concentration in culture supernatants was determined using an enzyme-linked immunosorbent assay (ELISA) kit (R&D Systems) as described by the manufacturer.

### High-throughput screening assay

Copanlisib- and duvelisib-resistant cells were seeded in 384-well plates at 500 cells per well and incubated with and without copanlisib (1 μM) or duvelisib (1 μM). After plating, cells were treated (in triplicate) with compounds from a kinase inhibitor library (1 μM; Selleck Chemicals) composed of 378 targeted agents that are included in clinical guidelines or in current clinical trials. After incubation at 37 °C in a humidified 5% CO_2_ incubator for 3 days, cell viability was analyzed using an ATP monitoring system based on firefly luciferase (ATPlite; PerkinElmer, Waltham, MA, USA). Ten of the most effective inhibitors were selected for combined treatment with copanlisib or duvelisib and compared with kinase inhibitor alone against drug-resistant cells.

### Antibodies for Western blotting

The antibodies employed included those specific for p-STAT3 (Y705) (rabbit monoclonal antibody, #9131), STAT3 (rabbit monoclonal antibody, #9139), p-STAT5 (Y694) (mouse monoclonal antibody, #9356), STAT5 (rabbit monoclonal antibody, #25656), p-AKT (S473) (rabbit monoclonal antibody, #4060), AKT (rabbit monoclonal antibody, #9272), p-p44/42 MAPK (T202/Y204) (rabbit polyclonal antibody, #9101), p44/42 MAPK (mouse monoclonal antibody, #9102), p-p70S6K (Thr389) (rabbit monoclonal antibody, #9234), p70S6K (rabbit polyclonal antibody, #9202), p-NF-κB (S536) (rabbit monoclonal antibody, #3033) and MCL-1 (rabbit monoclonal antibody, #4572), HRP-conjugated horse anti-mouse IgG (#7076) obtained from Cell Signaling (Beverly, MA), and NF-κB (mouse monoclonal antibody, sc-8008), BCL-xL (rabbit polyclonal antibody, sc-7195) and BCL-2 (mouse monoclonal antibody, sc-509), HRP-conjugated goat anti-rabbit IgG (sc-2004) obtained from Santa Cruz Biotechnology; β-actin (mouse monoclonal antibody, A5441) (Sigma St. Louis, MO, USA) was used as a loading control. All primary antibodies were diluted to 1:1000, and secondary antibodies were diluted to 1:3000.

### Apoptosis assay

Apoptosis was detected using an Annexin-V-fluorescein isothiocyanate (FITC) Apoptosis Detection Kit (BD Biosciences, San Jose, CA, USA) and a BD FACSVerse flow cytometry system (BD Biosciences). Parental and resistant cells were exposed to copanlisib or duvelisib, then harvested and processed according to the manufacturer’s instructions. Caspase-3/7 enzymatic activity was measured using a Caspase-Glo 3/7 Assay kit (Promega, Madison, WI, USA) according to the manufacturer’s instructions.

### Soft agar colony formation

Soft agar colony-formation assays were performed by first seeding cells in six-well plates (1 × 10^4^ cells/well) in a layer of 0.4% agar-RPMI-FBS over a bottom layer of 0.8% agar-RPMI-FBS. Cultures were maintained at 37 °C. On day 14, cells were fixed with pure ethanol containing 0.05% crystal violet, and colony formation efficiency was quantified by counting colonies containing at least 50 cells. OCI-Ly1 cells were seeded in six-well plates (500 cells/well) in triplicate in MethoCult H4100 (Stem Cell Technologies, Vancouver, British Columbia) for 14 days. Thereafter, colonies were counted and images acquired with the Gel Doc XR+ system (Bio-Rad, Hercules, CA).

## Results

### IL-6 expression is increased in B- and T-cell lymphoma cell lines with acquired resistance to copanlisib or duvelisib, respectively

**Fig. 1:**
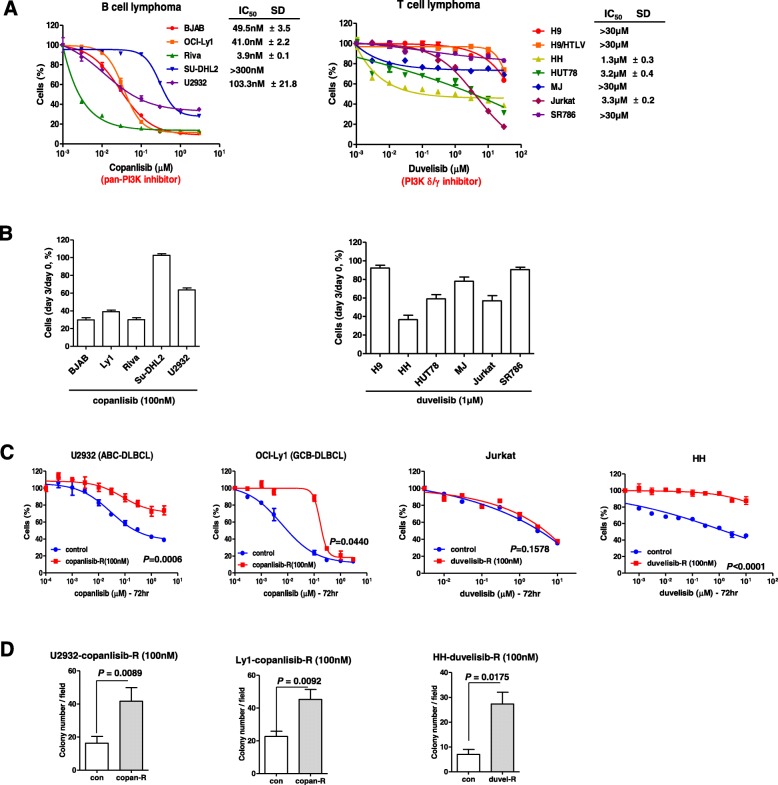
Copanlisib-resistant B-cell lymphoma cell lines and a duvelisib-resistant T-cell lymphoma cell line were established by long term exposure to low concentrations of the respective agents. **a** Concentration-response curves for copanlisib and duvelisib in B- and T-cell lymphoma cell lines. Cells were treated with the indicated concentrations of copanlisib or duvelisib for 72 h, and subjected to CCK-8 assays. **b** The cell growth percentage was evaluated by comparing live cell numbers on days 0 and 3 after treatment with copanlisib (100 nM) or duvelisib (1 μM). Viable cells were counted after trypan blue staining. **c**, **d** Copanlisib- or duvelisib-resistant B- and T-cell lymphoma cell line were established by long-term exposure to the corresponding agents. Copanlisib and duvelisib resistance were confirmed using CCK-8 assays (**c**) and colony-forming assays (**d**). Data represent means ± standard deviation of triplicate values. *P*-values were determined by Student’s t-test

**Fig. 2:**
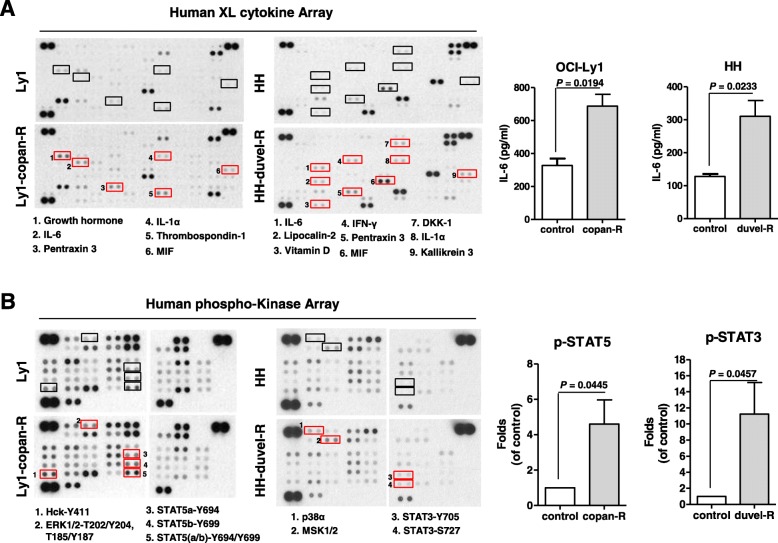
Induction of IL-6 and STAT3/5 in cells resistant to copanlisib or duvelisib. Cytokine (**a**) and phospho-kinase (**b**) expression in Ly1-copanlisib resistant cells, HH-duvelisib resistant cells, and parental cells. Individual cytokines and phospho-kinases were spotted in duplicate; induced molecules are indicated and labeled (red squares). Positive control spots are located at the corners of the human cytokine array and phospho-kinase array. ELISA results indicate IL-6 protein levels in culture supernatants. p-STAT3/5 was measured by Western blot analysis and normalized to STAT3/5. Data represent mean values ± SEM of three independent experiments. Each experiment was performed with triplicate samples. *P*-values were determined by Student’s t-test

Copanlisib and duvelisib have been shown to be effective in treating relapsed or refractory B-and T-cell lymphoma [8, 9, 18]. To study the molecular mechanism by which acquired resistance to PI3K inhibitor emerges, we exposed lymphoma cell lines to the PI3K inhibitors, copanlisib and duvelisib. Cell viability was assessed by treating B-cell lymphoma cell lines (BJAB, OCI-Ly1, Riva, SU-DHL2 and U2932) with copanlisib and T-cell lymphoma cell lines (H9, H9/HTLV, HH, HUT78, MJ, Jurkat and SR786) with duvelisib for 72 h. Riva, BJAB, OCI-Ly1 and U2932 B-cell lymphoma cells were sensitive to copanlisib, whereas Jurkat, HUT78 and HH T-cell lymphoma cell lines were found to be sensitive to duvelisib (Fig. 1a). To confirm that PI3K inhibitors actually suppressed the growth of the analyzed lymphoma cell lines, we evaluated the cell growth percentage by comparing viability at day 0 with that at day 3. Figure 1a & b shows that copanlisib and duvelisib decreased the growth of sensitive B- and T-cell lymphoma cell lines, respectively. To establish cells with acquired resistance, we chronically treated these sensitive cell lines with low concentrations of copanlisib or duvelisib. As shown in Fig. 1c, this approach led to the establishment of B-cell lymphoma cell lines with acquired resistance to copanlisib (U2932-copan-R and Ly1-copan-R) and a T-cell lymphoma cell line with acquired resistance to duvelisib (HH-duvel-R). The resultant resistant cell lines exhibited superior colony-forming ability (Fig. 1d). To elucidate the underlying mechanism of resistance, we analyzed differences in pathways and cytokine production between parental cells and their resistant counterparts. Cytokine array analyses showed that IL-6, IL-1α, and pentraxin 3 were increased in common in copanlisib-resistant Ly1-copan-R cells and duvelisib-resistant HH-duvel-R cells (Fig. 2a). An analysis of phospho-kinase arrays showed that p-STAT5(a/b)(Y694/Y699) was increased in copanlisib-resistant Ly1-copan-R cells, and p-STAT3(Y705/S727) was increased in duvelisib-resistant HH-duvel-R cells (Fig. 2b). On the basis of these results, we selected IL-6, a cytokine associated with STAT3 and STAT5 overexpression, for further analysis [19–21]. To provide a quantitative estimate of the IL-6 increase, we used an IL-6 ELISA kit. IL-6 production was increased 2–3-fold in resistant cells compared with control cells. No commonly expressed molecules were detected in the U2932-copanlisib resistant cell line, which was thus excluded from this experiment.

### IL-6 induces drug resistance by regulating STAT3/5

**Fig. 3:**
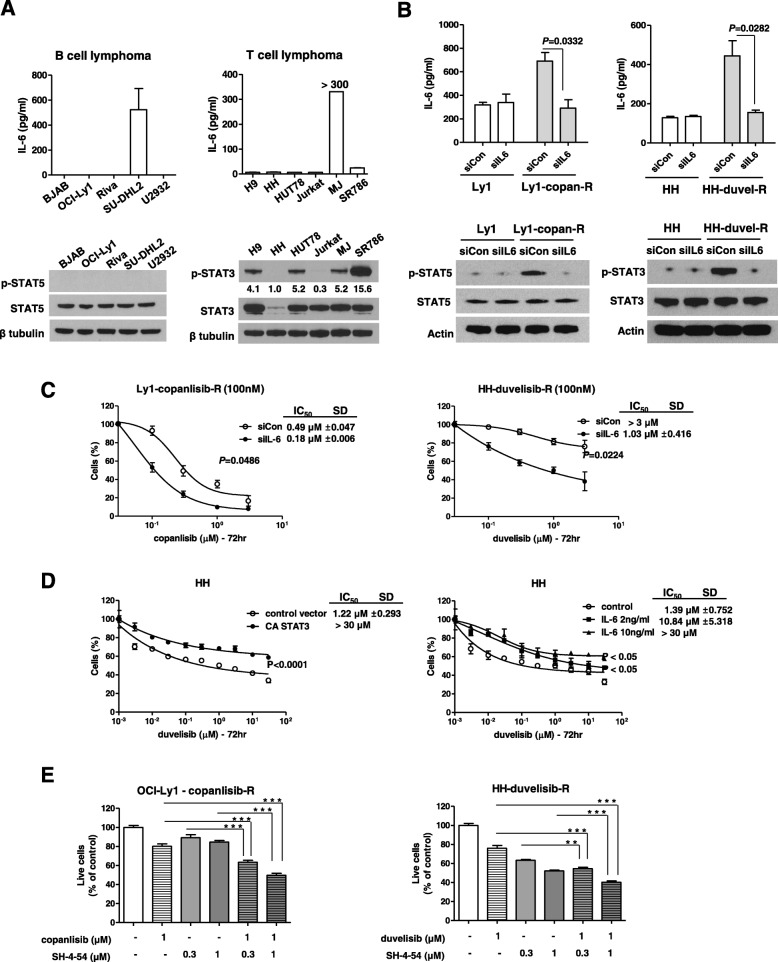
IL-6 is associated with activation of STAT3/5 and resistance to PI3K inhibitors. **a** Cytokine expression and phospho-kinase levels in parental B/T-cell lymphoma cell lines were examined by ELISA and Western blot analysis, respectively. The bands on the Western blots were quantified via densitometry. **b** Decreased IL-6 production was monitored by ELISA. STAT5 and STAT3 expression were examined by Western blot analysis; β-actin was included as a loading control. **c** IL-6 knockdown sensitized cell lines with acquired resistance to copanlisib or duvelisib. Resistant and parental lymphoma cells were transfected with siIL-6 or control siRNA. Cells were transiently transfected with siRNAs and then treated with copanlisib or duvelisib for 72 h, after which viability was assessed by CCK-8 assay. **d** Constitutively active STAT3 or IL-6 induced duvelisib resistance. Cells were transiently transfected with an expression plasmid for constitutively active STAT3 (STAT3-CA) or with a control vector. Cells were pre-treated with IL-6 (2 or 10 ng/ml) 2 h before duvelisib treatment. Median inhibitory concentration (IC_50_) values were calculated using GraphPad Prism 5 software (GraphPad). Data represent mean values ± SD of three independent experiments. **e** STAT inhibitor-sensitized lymphoma cells with acquired resistance to copanlisib or duvelisib. OCI-Ly1-copanlisib and HH-duvelisib resistant cells were treated with copanlisib (1 μM) or duvelisib (1 μM) in the presence or absence of SH-4-54 for 72 h. Cell viability was evaluated by trypan blue staining. *P*-values were determined by Student’s t-test and one-way repeated-measures ANOVA. Triple asterisk indicates statistically significant difference at *P* ≤ 0.005, double asterisk significant at *P* ≤ 0.01

We next examined whether IL-6 or phospho-STAT3/5 is upregulated in the resistant cell lines. Notably, IL-6 was upregulated in resistant cells compared with sensitive B and T cells (Fig. 3a). As shown in the figure, p-STAT3 was upregulated in resistant T cells but p-STAT5 was undetectable in resistant B cells. Next, we explored whether IL-6 expression modulates a relevant signaling pathway. siRNA-mediated knockdown of IL-6 in resistant cells was confirmed by IL-6 ELISA. Notably, IL-6 knockdown decreased STAT3 and STAT5 phosphorylation in Ly1-copan-R and HH-duvel-R cells, respectively (Fig. 3b), and sensitized resistant cells (Fig. 3c). To ascertain the influence of endogenous IL-6 production on cell proliferation, we incubated MJ cells with a neutralizing anti-IL-6 antibody (20 ng/ml). As shown in Additional file 1: Figure S1, comparison between untreated and treated cultures of MJ cells demonstrated that the cell number was lower in anti-IL-6 antibody treated cells (Additional file 1: Figure S1). To confirm that the underlying mechanism specifically involves IL-6/STAT3, we examined whether addition of IL-6 or ectopic expression of constitutively active STAT3 (STAT3-CA) alleviated the growth inhibition caused by PI3K inhibitors. As shown in Fig. 3d, IL-6 and STAT3-CA increased duvelisib resistance. We also examined the effect of SH-4-54 (a STAT inhibitor) combined with copanlisib or duvelisib on cell viability in resistant cell lines (Fig. 3e). The combination of the STAT inhibitor with copanlisib or duvelisib was shown to lead to a significant decrease in the viability of resistant cells. These results collectively indicate that IL-6/STAT3 activation is critical for the development of resistance to the PI3K inhibitor in lymphoma.

### Inhibition of JAK sensitizes resistant cells to copanlisib or duvelisib

**Fig. 4:**
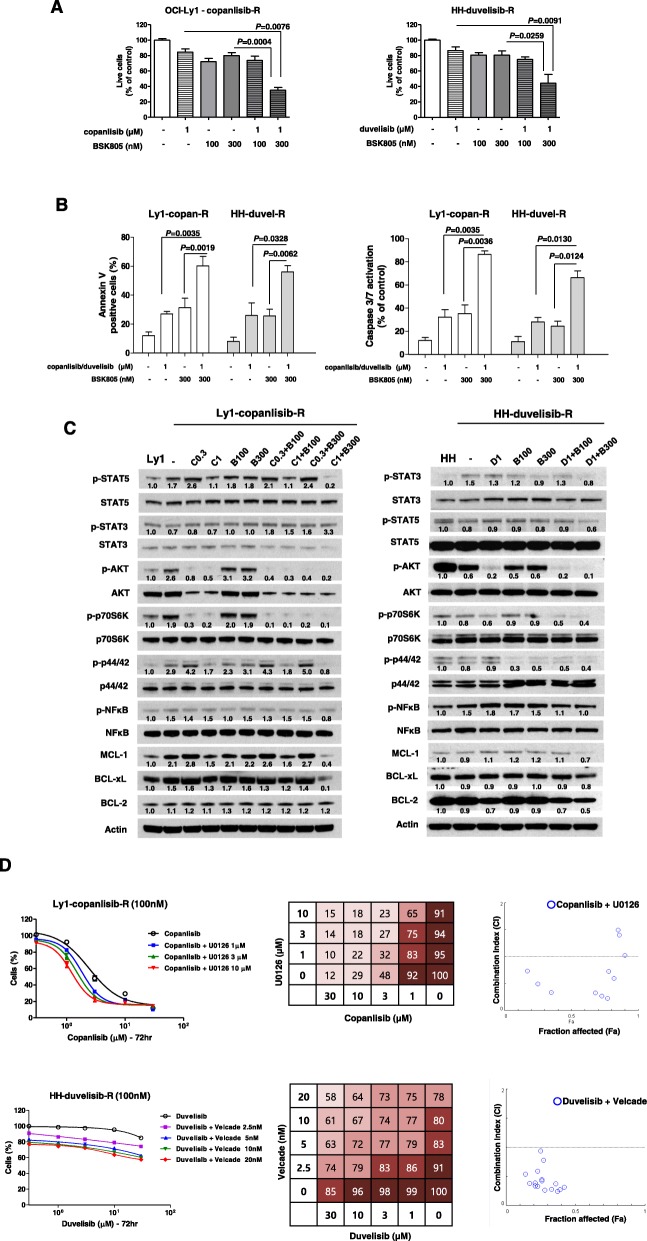
Combined treatment of resistant lymphoma cells with BSK805 (and other targeted drugs) with copanlisib or duvelisib. **a** OCI-Ly1-copanlisib and HH-duvelisib resistant cells were treated with copanlisib (1 μM) or duvelisib (1 μM) in the presence or absence of BSK805 (100 and 300 nM) for 72 h. Cell viability was evaluated by trypan blue staining. b Apoptosis was detected using annexin V/propidium iodide staining. Caspase-3/7 enzymatic activity was measured using a luminometer. Data represent mean values ± SEM of three independent experiments. Each experiment was performed with triplicate samples. *P*-values were determined by one-way repeated-measures ANOVA. Triple asterisk indicates statistically significant difference at *P* ≤ 0.005, double asterisk significant at *P* ≤ 0.01. **c** Western blot analysis of STAT3, STAT5, AKT, MAPK, NF-κB, and p70S6K phosphorylation in resistant cells treated with copanlisib (0.3 and 1 μM) or duvelisib (1 μM) and BSK805 (100 and 300 nM). The bands on the Western blots were quantified via densitometry. **d** Responses to single agents and combined treatment regimens were evaluated by CCK-8 assay and isobologram analysis. Viability, estimated by CCK-8 assay, was determined in matrix block experiments. Interactions of copanlisib and U0126 or duvelisib and velcade were assessed by determining combination index (CI) values using CalcuSyn software (Biosoft, Ferguson, MO, USA), where CI values > 1, = 1 and < 1 signify antagonism, additivity and synergy, respectively

To identify inhibitors that are effective in treating PI3K inhibitor-resistant cells in combination with copanlisib or duvelisib, we screened 378 kinase inhibitors using a high-throughput assay. Of these inhibitors, 10 selected as effective upon co-administration with copanlisib or duvelisib were shown to lead to a significant decrease in the viability of resistant cells (Additional file 1: Figure S2a). The combination of a PI3K inhibitor with a MEK inhibitor (refametinib or U0126) was effective in the Ly1-copan-R cell line, and Aurora kinase inhibitors (KW-2449, CCT129202 and SNS 314 mesylate) were effective in HH-duvel-R cells. Interestingly, the JAK inhibitor, BSK805, effectively inhibited both Ly1-copan-R and HH-duvel-R cells (Additional file 1: Figure S2a). Among the kinase inhibitors listed in the table, BSK805, PFK15, KW2449 and AZD1080 exhibited synergistic growth-inhibitory effects in combination with copanlisib or duvelisib in resistant cells (Fig. 4a, Additional file 1: Figure S2b). Co-treatment with BSK805 and copanlisib or duvelisib induced apoptosis, as evidence by Annexin V positivity and caspase 3/7 activation (Fig. 4b). Next, we examined the effect of combination treatment on signaling pathways in resistant cells. As shown in Fig. 4c, p-STAT5, p-AKT, p-p70S6K, p-p44/42 and MCL-1 were increased in the Ly1-resistant cell line. Simultaneous treatment of these cells with BSK805 and copanlisib significantly reduced p-STAT5 and p-p44/42 levels, and also suppressed expression of MCL-1 and BCL-xL. On the other hand, combination treatment with duvelisib and BSK805 increased p-STAT3 and p-NF-κB and decreased MCL-1, BCL-xL and BCL-2 in the HH-resistant cell line. We also examined the effect of U0126 (p-p44/42 inhibitor) or velcade (p-NF-κB inhibitor) combined with copanlisib or duvelisib on lymphoma cell viability (Fig. 4d). This analysis showed that U0126 or velcade together with copanlisib or duvelisib were also effective in Ly1-copan-R or HH-duvel-R cells. Collectively, these results suggest that acquired copanlisib or duvelisib resistance is also associated with alternative activation of AKT/mTOR/MAPK or NF-κB pathways.

## Discussion

Recent studies have suggested several possible mechanisms that may explain the resistance of cells to PI3K inhibitors, including amplification/mutation of PIK3CA and PIK3CB, loss of PTEN, and changes in tumor microenvironment. Moreover, activation of alternative pathways (WNT/β-catenin, ERK/MAPK, JAK/STAT, NOTCH) is known to be a major cause of therapeutic resistance to molecular-target inhibitors [22]. ERK-dependent IL-6 signaling can form a positive feedback loop that compensates for AKT inactivation and is closely associated with adaptive resistance [23]. Combining MEK1/2 inhibitors with BKM120 was shown to enhance the anti-tumor effects of BKM120 [24]. Combined treatment with ibrutinib and a p110α inhibitor may be a promising therapeutic strategy to overcome stromal cell-mediated ibrutinib resistance in MCL [25]. Combination of ibrutinib with the PI3Kα/δ inhibitor, copanlisib, produced a sustained complete response in vivo in CD79Bmut/MYD88mut ABC-DLBCL models [26].

In this study, we sought to identify an alternative pathway that is responsible for resistance to the PI3K inhibitors, copanlisib and duvelisib, in B- and T-cell lymphoma. Using cytokine array analyses, we found that IL-6 was increased in PI3K inhibitor-resistant B- and T-cell lymphoma cells, and showed that the acquired resistance to copanlisib and duvelisib reflected two mechanisms in common: upregulation of IL-6 and activation of STAT3/5. A previous report [18] showed that chemokines CCL11 and CXCL13 and cytokine IL-17a are involved in the response to duvelisib. However, our arrays showed no significant changes in these chemokines/cytokines, suggesting that the profile of duvelisib-responsive chemokines/cytokines may differ depending on the cell subtype. Resistant cultures may exhibit greater aggressiveness compared with the initial cell population owing to increased levels of IL-6 released into the microenvironment. An increase in IL-6 levels could reflect an increase in the cytokine IL-1β or the chemokine CXCL8. However, we found no increase in IL-1β or CXCL8 in resistant cultures; accordingly, local inflammation was not considered as a possible side effect. Interestingly, a cytokine array revealed that IL-1α, pentraxin-3, and MIF were commonly expressed in resistant cells. IL-1α is known to induce IL-6 secretion and increase invasiveness, immune suppression, survival and proliferation [27]. PTX3, which is encoded by an IL-1-inducible gene, binds to apoptotic cells and regulates their clearance by antigen-presenting dendritic cells [28]. MIF (macrophage migration inhibitory factor) is an inflammatory cytokine that binds to the CD74 receptor complex to trigger ERK phosphorylation [29]. We also observed increased phosphorylation of ERK1/2, HCK (hematopoietic cell kinase), p38α and MSK1/2 in resistant cells, as assessed by phosphor-kinase array analysis. HCK is a member of the SRC family of cytoplasmic tyrosine kinases and enhances cell proliferation and survival by physically associating with oncogenic proteins. HCK activation can also reduce drug efficacy and contribute to chemoresistance. HCK is activated by various stimuli, including IL-6 [30], and conversely regulates IL-6 production [31]. MSK1 (mitogen- and stress-activated kinase 1) is a serine/threonine kinase that is activated by ERK and p38 MAPK [32]. Although a number of cell lines were used herein, this study is limited by its lack of experiments involving in vivo animal models or clinical specimens.

Previous reports have used combined inhibition of activated STAT signaling pathways and IL-6 as a therapeutic approach against drug-resistant cells, and other studies have shown that Taxol resistance in ovarian cancer can be overcome by inhibiting IL-6 and STAT3 [33, 34]. STAT3 and the highly homologous isoforms STAT5A and STAT5B (STAT5A/B) are key components of the Janus tyrosine kinase (JAK)/STAT pathway [35]. Previous studies have shown that STAT3 and STAT5 can serve as therapeutic targets [36], and their expression is a predictive biomarker of drug resistance in cancers [37]. In breast cancer, JAK2/STAT5 activation is associated with resistance to PI3K/mTOR inhibitors, and combination therapy targeting JAK2/STAT5 and PI3K/mTOR was proposed to overcome this resistance [38].

To explore potential drugs that are effective against PI3K inhibitor (copanlisib and duvelisib)-resistant B- and T-cell lymphomas, we used high-throughput screening assays, which led to the identification of the JAK inhibitor, BSK805. We found that combined treatment with BSK805 and a PI3K inhibitor exerted a synergistic effect. Interestingly, ruxolitinib, which is clinically used as a JAK inhibitor, was not effective in inhibiting these PI3K inhibitor-resistant cell lines. On the basis of these findings, we speculate that activation of IL-6 signaling may contribute to the PI3K resistance of B- and T-cell lymphoma cells. Given that MAPK and NF-κB signaling is increased in resistant cells, we investigated the effect of the corresponding inhibitors, U0126 and velcade, in these resistant cell lines. MAPK and NF-κB, which remain active in IL-6–high lymphoma cells, may promote continued survival and growth, even upon PI3K inhibition. The synergistic effects of a PI3K inhibitor (copanlisib or duvelisib) with U0126 or velcade support the therapeutic potential of these drug combinations in IL-6-high resistant cases.

Data from the current study indicate that PI3K inhibition with copanlisib or duvelisib may not be sufficient to suppress the entire PI3K downstream signaling pathway in B- and T-cell lymphoma. PI3K inhibitor treatment selectively antagonizes activation of AKT and p70S6K. However, our data show that survival of lymphoma cells with activated STAT3/5, MAPK, and NF-κB is maintained. STAT3/5, MAPK, and NF-κB inhibitors could bypass various gain-of-function upstream mutations in the receptor-mediated signaling pathway.

## Conclusions

The current findings provide preliminary evidence of the functional consequences of prolonged treatment with copanlisib and duvelisib in B- and T-cell lymphoma, and reinforce the importance of combination therapy for copanlisib- and duvelisib-resistant lymphoma.

## Additional file

Additional file 1: **Figure S1.** Effects of a neutralizing anti-IL-6 antibody on duvelisib-resistant MJ cells. Cells were pre-incubated with 20 ng/ml monoclonal rat anti-human IL-6 antibody for 2 h and then treated with duvelisib for 72 h, after which viability was assessed by CCK-8 assay. Data represent the mean of triplicate samples in a representative experiment. *P*-values were determined by the Student’s t-test. **Figure S2.** Screening for kinase inhibitors that synergistic effect with copanlisib or duvelisib in resistant cell lines. **a** Copanlisib- and duvelisib-resistant cells, with and without treatment with copanlisib (1 μM) or duvelisib (1 μM), were co-treated with compounds from a kinase inhibitor library (1 μM; *n* = 3 for each condition) for 72 h. Responses to single agents (kinase inhibitor) and combined treatment regimens (kinase inhibitor with either copanlisib or duvelisib) were evaluated using an ATP monitoring system based on firefly luciferase. The 10 most effective inhibitors were selected. **b** HH-duvelisib resistant cells were treated with copanlisib (1 μM) or duvelisib (1 μM) in the presence or absence of PFK15, KW2449 and AZD1080 (100 and 300 nM) for 72 h. Cell viability was evaluated by trypan blue staining. P-values were determined by one-way repeated-measures ANOVA. Triple asterisk indicates statistically significant difference at *P* ≤ 0.005, double asterisk significant at *P* ≤ 0.01. (DOCX 237 kb)

## Data Availability

All data generated or analyzed during this study are included in this published article.

## Abbreviations

ABC DLBCL: Activated B cell-like diffuse large B-cell lymphoma
BCR: B-cell receptor
CLL: Chronic lymphocytic leukemia
CXCL: Chemokine ligand
ERK: Extracellular signal-regulated kinase
IL-6: Interleukin-6
JAK: Janus kinase
MAPK: Mitogen-activated protein kinase
mTOR: Mammalian/mechanistic target of rapamycin
NF-κB: Nuclear factor kappa-light-chain-enhancer of activated B cells
NK: Natural killer
p70S6K: Ribosomal protein S6 kinase beta-1
PI3K: Phosphoinositol 3-kinase
PIK3CA: Phosphatidylinositol 4,5-bisphosphate 3-kinase catalytic subunit alpha isoform
PIK3CB: Phosphatidylinositol 4,5-bisphosphate 3-kinase catalytic subunit beta isoform
PTEN: Phosphatase and tensin homolog
STAT: Signal transducer and activator of transcription
