# Supplementary material for: Interleukin-6 mediates resistance to PI3K-pathway–targeted therapy in lymphoma
Source: BMC Cancer. 2019 Oct 10;19:936. doi: 10.1186/s12885-019-6057-7 (PMC6785854; doi:10.1186/s12885-019-6057-7)
Supplement: Supplementary file 1 — Figure S1. Effects of a neutralizing anti-IL-6 antibody on duvelisib-resistant MJ cells. Cells were pre-incubated with 20 ng/ml monoclonal rat anti-human IL-6 antibody for 2 h and then treated with duvelisib for 72 h, after which viability was assessed by CCK-8 assay. Data represent the mean of triplicate samples in a representative experiment. P-values were determined by the Student’s t-test. Figure S2. Screening for kinase inhibitors that synergistic effect with copanlisib or duvelisib in resistant cell lines. a Copanlisib- and duvelisib-resistant cells, with and without treatment with copanlisib (1 μM) or duvelisib (1 μM), were co-treated with compounds from a kinase inhibitor library (1 μM; n = 3 for each condition) for 72 h. Responses to single agents (kinase inhibitor) and combined treatment regimens (kinase inhibitor with either copanlisib or duvelisib) were evaluated using an ATP monitoring system based on firefly luciferase. The 10 most effective inhibitors were selected. b HH-duvelisib resistant cells were treated with copanlisib (1 μM) or duvelisib (1 μM) in the presence or absence of PFK15, KW2449 and AZD1080 (100 and 300 nM) for 72 h. Cell viability was evaluated by trypan blue staining. P-values were determined by one-way repeated-measures ANOVA. Triple asterisk indicates statistically significant difference at P ≤ 0.005, double asterisk significant at P ≤ 0.01. (DOCX 237 kb) [file 12885_2019_6057_MOESM1_ESM.docx]

**Figure S1.**

**
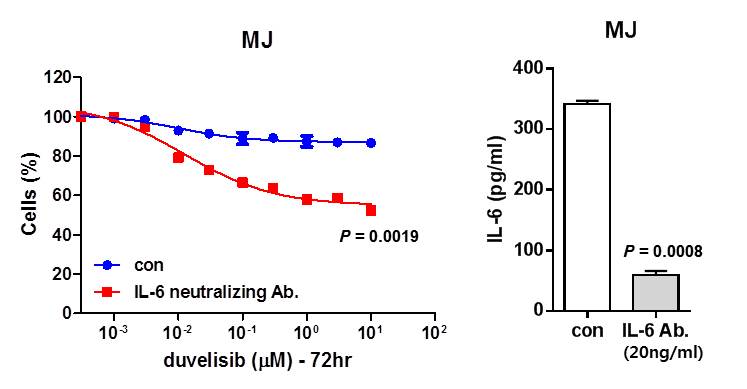
**

**Figure S2.**

**
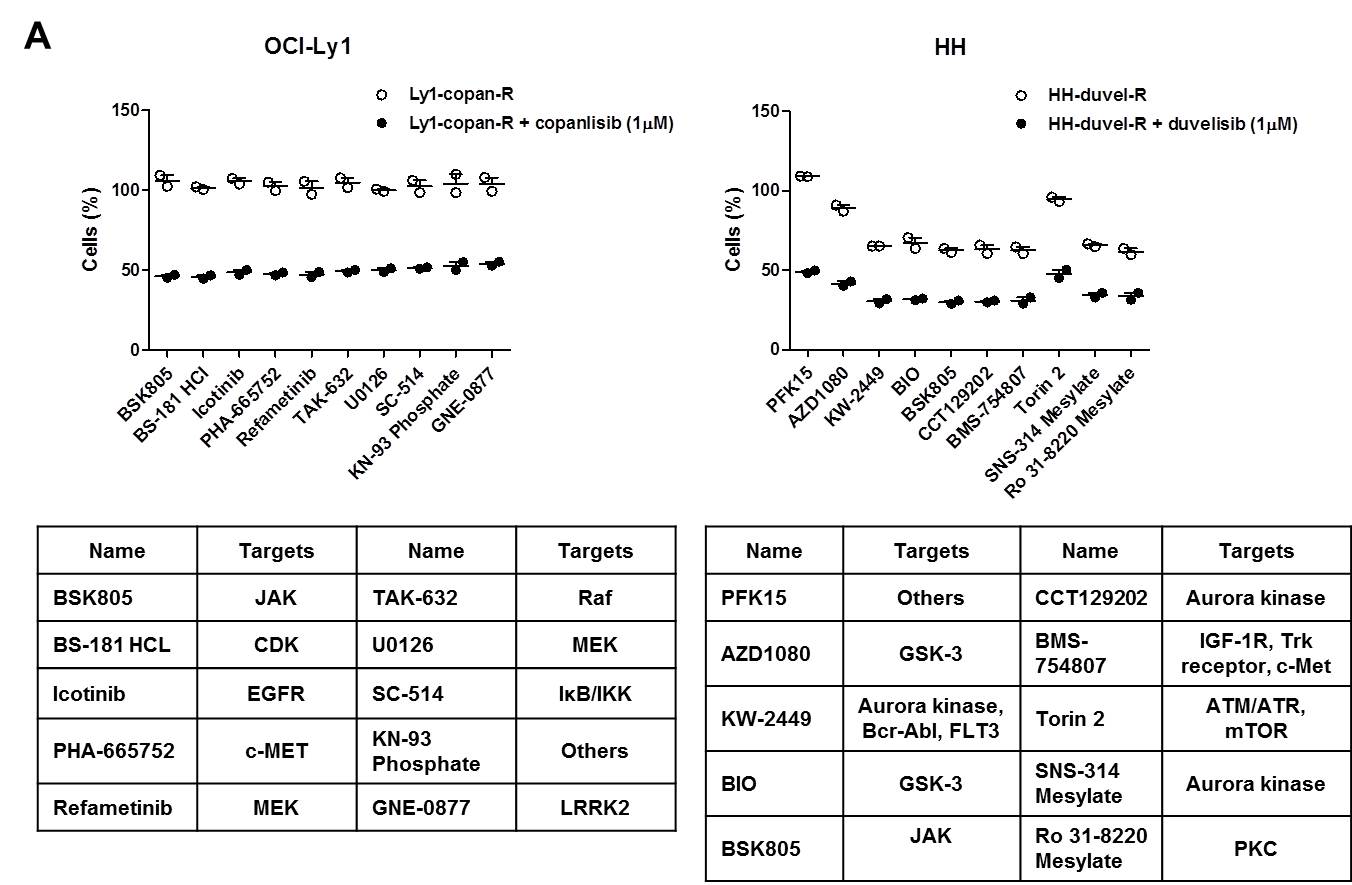
**

**
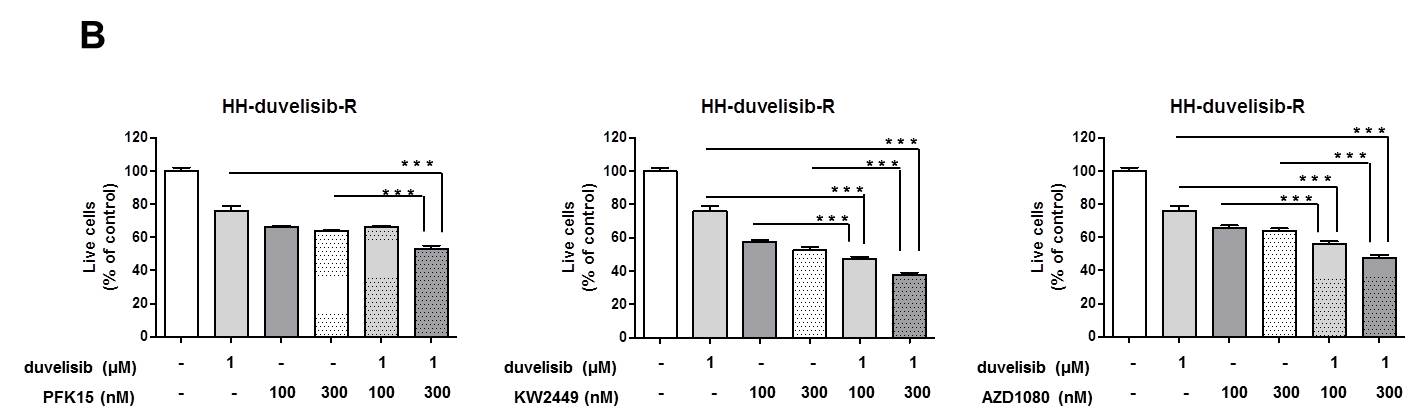
**

**Figure S1.** Effects of a neutralizing anti-IL-6 antibody on duvelisib-resistant MJ cells. Cells were pre-incubated with 20 ng/ml monoclonal rat anti-human IL-6 antibody for 2 h and then treated with duvelisib for 72 h, after which viability was assessed by CCK-8 assay. Data represent the mean of triplicate samples in a representative experiment. P-values were determined by the Student’s t-test.

**Figure S2.** Screening for kinase inhibitors that synergistic effect with copanlisib or duvelisib in resistant cell lines. **a** Copanlisib- and duvelisib-resistant cells, with and without treatment with copanlisib (1 μM) or duvelisib (1 μM), were co-treated with compounds from a kinase inhibitor library (1 μM; n = 3 for each condition) for 72 h. Responses to single agents (kinase inhibitor) and combined treatment regimens (kinase inhibitor with either copanlisib or duvelisib) were evaluated using an ATP monitoring system based on firefly luciferase. The 10 most effective inhibitors were selected. **b** HH-duvelisib resistant cells were treated with copanlisib (1 μM) or duvelisib (1 μM) in the presence or absence of PFK15, KW2449 and AZD1080 (100 and 300 nM) for 72 h. Cell viability was evaluated by trypan blue staining. P-values were determined by one-way repeated-measures ANOVA. Triple asterisk indicates statistically significant difference at P ≤ 0.005, double asterisk significant at P ≤ 0.01.
